# The Cause of Alzheimer’s Disease: The Theory of Multipathology Convergence to Chronic Neuronal Stress

**DOI:** 10.14336/AD.2021.0529

**Published:** 2022-02-01

**Authors:** Boris Decourt, Gary X D’Souza, Jiong Shi, Aaron Ritter, Jasmin Suazo, Marwan N Sabbagh

**Affiliations:** ^1^Translational Neurodegenerative Research Laboratory, Cleveland Clinic Lou Ruvo Center for Brain Health, Las Vegas, NV 89106, USA.; ^2^Department of Neurological Sciences, Rush University Medical Center, Chicago, IL, USA.; ^3^Cleveland Clinic Nevada and Lou Ruvo Center for Brain Health, Las Vegas, NV 89106, USA.

**Keywords:** Chronic Neuronal Stress, Convergence, Hypoxia, Inflammation, Mitochondria, Multipathology, Oxidative Stress, Starvation

## Abstract

The field of Alzheimer’s disease (AD) research critically lacks an all-inclusive etiology theory that would integrate existing hypotheses and explain the heterogeneity of disease trajectory and pathologies observed in each individual patient. Here, we propose a novel comprehensive theory that we named: the multipathology convergence to chronic neuronal stress. Our new theory reconsiders long-standing dogmas advanced by previous incomplete theories. Firstly, while it is undeniable that amyloid beta (Aβ) is involved in AD, in the seminal stage of the disease Aβ is unlikely pathogenic. Instead, we hypothesize that the root cause of AD is neuronal stress in the central nervous system (CNS), and Aβ is expressed as part of the physiological response to protect CNS neurons from stress. If there is no return to homeostasis, then Aβ becomes overexpressed, and this includes the generation of longer forms that are more toxic and prone to oligomerization. Secondly, AD etiology is plausibly not strictly compartmentalized within the CNS but may also result from the dysfunction of other physiological systems in the entire body. This view implies that AD may not have a single cause, but rather needs to be considered as a spectrum of multiple chronic pathological modalities converging to the persistent stressing of CNS neurons. These chronic pathological modalities, which include cardiovascular disease, metabolic disorders, and CNS structural changes, often start individually, and over time combine with other chronic modalities to incrementally escalate the amount of stress applied to CNS neurons. We present the case for considering Aβ as a marker of neuronal stress in response to hypoxic, toxic, and starvation events, rather than solely a marker of AD. We also detail numerous human chronic conditions that can lead to neuronal stress in the CNS, making the link with co-morbidities encountered in daily clinical AD practice. Finally, we explain how our theory could be leveraged to improve clinical care for AD and related dementia in personalized medicine paradigms in the near future.

## 1. Introduction

Alzheimer’s disease (AD) is the most prominent type of dementia. This neurodegenerative disorder affects an estimated 50 million individuals worldwide, and this figure is expected to triple by 2050 [1]. The current annual financial cost of AD care in the United States alone is estimated to be $300 billion [1]. Furthermore, many family members of affected patients are forced to halt their professional and social activities to become caregivers, adding to the toll of this debilitating disease on human societies. Taken together, these figures highlight the urgent need to develop safe and effective therapeutic interventions to alter the trajectory of this disease. However, an incomplete understanding of the complex etiology of AD makes it difficult to prevent its onset and/or significantly slow its progression. Overall, after factoring multiple parameters such as the number of trained neurologists, AD affects the population of all countries at a similar rate [1], which hinders the pinpointing of discrete genetic or environmental risk and protective factors outside those already known, such as age and Apolipoprotein E (ApoE) allelic status. Nonetheless, the early onset of symptoms spotted in singular families and geographical areas has allowed for the identification of critical genetic mutations on three key genes that accelerate or slow down the disease course of AD. For instance, the Iceland mutation on the amyloid precursor protein (APP) at position 673 (A673T) was shown to decrease the incidence of AD in carriers of this variant [2]. On the other hand, over 68 mutations in *APP*, 300 in *Presenilin 1* (PSEN1), and 64 in *Presenilin 2* (PSEN2) (www.alzforum.org/mutations), as well as the triplication of chromosome 21 (Down syndrome), which bears the *APP* gene, often results in increased production of longer forms of the amyloid beta (Aβ) peptides that are more prone to oligomerization, leading to an early onset of AD. However, genetic forms of AD represent only around 5% of all cases, with 95% of AD cases being considered sporadic with undefined causes [3].

The pathological hallmarks of AD in the central nervous system (CNS) are: (i) the extracellular accumulation of APP-derived Aβ oligomers and other materials into dense senile plaques; (ii) the intraneuronal hyperphosphorylation of the microtubule-binding protein tau which induces its aggregation into neurofibrillary tangles (NFTs); and (iii) chronic inflammation [4]. The combination of these complex and overlapping pathologies induce synaptic loss and neuronal death, which further exacerbates each pathology [5], hence forming a chronic feedback loop that can’t be arrested.

Strikingly, AD seems to exclusively affect humans. Analysis of animal brain tissue has led to the observation that only polar bears and a few wild felines naturally display the neuropathological hallmarks of AD, i.e. Aβ plaques and hyperphosphorylated tau, though rarely in the form of NFTs [6, 7]. However, in their natural niche animals do not exhibit the full range of AD cognitive and neuropsychiatric symptoms noted in humans [7, 8]. Thus, it appears that conditions specific to humans are causing AD. Such conditions could be multifactorial, such as: (i) the sequence of Aβ in most animals is different from humans, especially for animals farther in the phylogenetic tree [7]; (ii) most animals have a tightly regulated homeostasis that prevents the development of chronic metabolic disorders like obesity (except domestic cats and dogs), thereby animals rarely or do not at all experience oxidative stress and inflammation events associated with such metabolic disorders; and (iii) animals suffering pathologies affecting their cognitive functions may be impaired in finding sufficient food, leading to a rapid death. These possible differences also highlight the issue of finding appropriate animal models to study the full range of human AD pathological and behavioral changes in laboratories [9]. Therefore, to understand the mechanisms that induce AD it is critical to focus on conditions unique to humans.

Over the past decades, several theories have been advanced to explain the etiology of AD, which can be divided into two main categories. First, some theories explain possible causes of AD, such as the cholinergic hypothesis, inflammation hypothesis, lymphatic system hypothesis, metal ion hypothesis, and vascular dysfunction hypothesis. Second, some theories focus more on the pathological events taking place after AD has started, and include the amyloid cascade hypothesis, calcium homeostasis hypothesis, mitochondrial cascade hypothesis, and the tau propagation hypothesis. Readers wanting to learn more about these theories are referred to a thorough summary published recently [10]. Among those, the vascular hypothesis is likely the most exhaustive today [elegantly reviewed in [11]. However, although each of these theories provides a pertinent reasoning for the specific mechanism they highlight, the field critically lacks a comprehensive meta-theory that would harmoniously integrate all previous AD hypotheses, as well as the voluminous clinical and pathophysiological observations reported in the literature. This comprehensive theory would have the following characteristics: (i) fully explain the complex nature of AD; (ii) improve the selection of patients and outcomes of clinical trials; and (iii) ameliorate AD healthcare in the near future.

In this opinion article, we describe an innovative, integrative theory that has the potential to explain the root cause of AD, as well as the reasons behind the heterogeneity in disease trajectory reported for AD. However, we will not describe in detail the molecular mechanisms leading to the prototypical AD neuropathologies, e.g., extracellular Aβ plaques and NFT, as these events occur later in the disease progression, thus are outside of the scope of the present etiology theory. We base our new theory on an extensive review of the scientific literature covering *in vitro* and laboratory animal models of AD, as well as human research.

## 2. The Theory of Multipathology Convergence to Chronic Neuronal Stress

**Figure 1.**
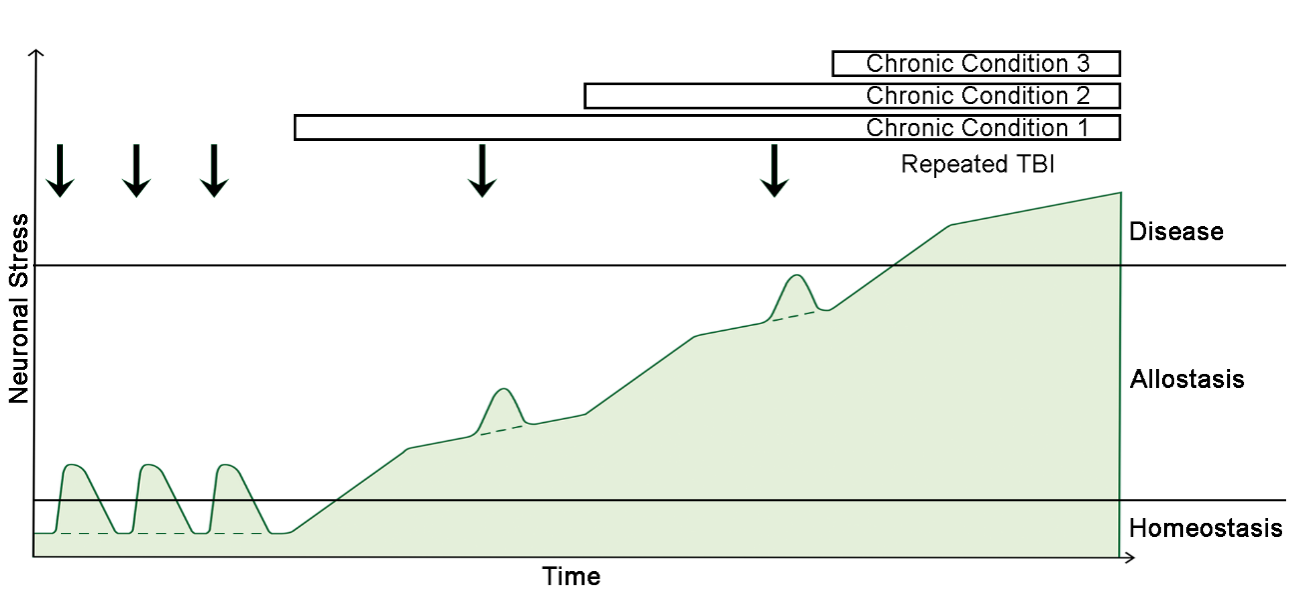
Schematic of the theory of multipathology convergence to neuronal stress leading to AD. In homeostasis, neuronal stress is low. Repeated traumatic brain injuries (TBI) may transiently increase neuronal stress in the CNS, but the levels of stress return back to homeostasis naturally. When a severe central or peripheral chronic condition occurs, the level of neuronal stress increases to an allostatic state, which may combine with TBI. Over time, additional chronic conditions stack up on top of the first condition to continually increase CNS neuronal stress up to the disease state. If there is no TBI in the life of a given patient, then the levels of neuronal stress follow the dashed line. Currently, there are no FDA-approved AD therapeutic intervention to guide the conversion from allostasis back to homeostasis, thus most patients progress to disease state.

We provocatively propose to re-think three crucial long-standing dogmas when contemplating AD. Firstly, while Aβ is undeniably involved in AD, in the seminal stage of the disease Aβ is unlikely pathogenic, but instead is expressed as part of the physiological response to protect CNS neurons from stressful events. Thus, we posit that neuronal stress is the root cause of AD, not Aβ. If there is no return to homeostasis, then Aβ becomes overexpressed. This includes the generation of longer peptides that are more prone to form multimers and fibrils, which are the most toxic forms of Aβ. Secondly, AD etiology is plausibly not strictly compartmentalized within the CNS. Instead, AD etiology may be impacted by the dysfunction of other physiological systems in the body that alter oxygen and nutrients delivery and utilization in the CNS, with a major impact on energy production. This is possibly combined with the impairment of waste removal and detoxification processes, as well as the deterioration of the regulatory systems controlling the levels and activities of reactive oxygen (e.g., superoxide radical anion O2^*-^, hydroxyl radical OH^*^, hydrogen peroxide H_2_O_2_) and nitrogen (e.g., nitric oxide NO^*^, nitrogen dioxide NO2^*^, peroxynitrite ONOO^-^/ONOOH) species (ROS and RNS). Thirdly, AD may not have a single cause, but rather needs to be considered as a spectrum of multiple chronic central and/or peripheral pathological modalities converging to the persistent stressing of CNS neurons. These chronic pathologies often start individually, and over time may combine with other chronic modalities to incrementally accrue the stress applied to CNS neurons (Fig. 1). When AD is considered in this manner, our new theory seamlessly depicts the mechanisms that precede the events described in the amyloid cascade hypothesis and the hypothetical model of changes in AD biomarkers [12]. In addition, our integrative theory supports the idea of a transition allostatic stage between normalcy and AD (Fig. 1), which is the current vision for the condition termed mild cognitive impairment (MCI) since it is not considered as a dementia stage [13]. Plus, given the fact that the combination of multiple chronic disorders can lead to neuronal stress, our new theory organically illustrates the numerous possibilities in rates of clinical decline and heterogeneity in disease trajectory and pathological features observed in individual AD patients. For example, a patient suffering sleep apnea and chronic liver disease will have a different disease trajectory than another patient experiencing a cardiovascular disease. Furthermore, the mingling of AD with other chronic pathologies would partly explain why it is proven difficult to identify AD-specific biomarkers in peripheral biofluids, since biomarkers for non-AD pathologies would largely mask AD-specific markers prior to the onset of advanced AD pathology and symptoms. Attractively, however, if some symptoms and/or biomarkers can be uncovered prior to the onset of dementia, e.g. at pre-symptomatic or prodomodal/MCI stages, then implementing personal medicine paradigms with changes in lifestyle and/or the environment may slow down or reverse conversion to AD. The logically built sections below provide the scientific rationale supporting our new disruptive theory on the root cause of AD.

## 3. Pathophysiology of Neuronal Stress

The identification of genetic mutations in isolated families suffering early onset of AD has led to the strong belief that Aβ is the single cause of AD. This conviction motivated the formulation of the amyloid cascade hypothesis [14]. However, intensive work over the past three decades has shown that Aβ is involved in numerous physiological functions in the entire body throughout life. Critically, in the brain, Aβ expression increases during acute and chronic stressing events, supporting the idea that monomeric Aβ could be part of an immune-like response in the CNS [15, 16], i.e., an early marker of central inflammation rather than the single cause of AD. In the sub-sections below, we describe the currently known physiological functions of Aβ and several stressors that generate an increase in Aβ expression in CNS neurons.

### 3.1. Amyloid Beta Metabolism and Physiology

#### 3.1.1. Amyloid Beta Synthesis

Amyloid beta peptides are generated via consecutive proteolysis of the single-pass transmembrane protein APP by β- and γ-secretases, which is referred to as the amyloidogenic pathway. APP has three major splicing variants of 695, 751, and 770 amino acids that can generate Aβ [17]. The main β-secretase in AD was identified as beta amyloid converting enzyme 1 (BACE1) [18]. BACE1 cleaves APP in the outer space near the plasma membrane and lysosomes [19] to release a large soluble extracellular fragment called sAPPβ. The remaining transmembrane domain, termed C99, is then minced by γ-secretase to produce Aβ peptides of 37-51 amino acids in length, and releases in the cytoplasm the APP intracellular domain (AICD), which is 48-53 amino acids in length and that can modulate transcription activities in the nucleus [20-22]. Gamma-secretase is a tetramer comprising PSEN1 or PSEN2, anterior pharynx-defective 1 (APH-1), nicastrin (NCT), and presenilin enhancer 2 (PEN-2) [23]. Mutations identified in *PSEN1* and *PSEN2* affect the length of Aβ peptides. The *PSEN1/2* mutations often increase the length of Aβ to 42 or more amino acids [22]. Not surprisingly, the senile plaques found in AD are rich in physiological Aβ40 and anomalous Aβ42 peptides.

A second possibility for APP proteolysis is the non-amyloidogenic pathway. In this case, APP is first cut by an α-secretase inside the Aβ sequence, precisely between Aβ amino acids 16 and 17, thereby releasing a large extracellular piece termed sAPPα, which is the same as sAPPβ plus the first 16 amino acids of Aβ. The leftover membrane-tethered C83 segment is then cleaved by γ-secretase to release a short, non-amyloid peptide called p3 (i.e., Aβ truncated from the first 16 amino acids) of unknown function, and AICD [21, 22].

APP is expressed by virtually all cells in the body throughout life. Most cells express the APP751/770 variants and process APP via the non-amyloidogenic pathway. However, in physiological circumstances some cells primarily process APP via the amyloidogenic pathway to produce Aβ peptides (principally Aβ40), including platelets and CNS neurons [24]. Interestingly, astrocytes and microglia can also produce high amounts of Aβ during CNS insults [25]. To note, neurons preferentially express APP695, while platelets, astrocytes, and microglia express APP751/770 variants [17]. Amyloid beta produced by neurons can either be secreted in the extracellular milieu or remain inside the cytoplasm where it interacts with intracellular proteins and is transported inside the mitochondrial matrix via sequential passage through the translocase of the outer mitochondrial membrane (TOM) and translocase of the inner membrane (TIM) complexes [26]. Other cells in the body, such as leukocytes, can regulate the amount of APP processed via the amyloidogenic and non-amyloidogenic pathways based on pathophysiological status [27].

Remarkably, Aβ is only produced from APP. Although they have a high degree of homology to APP, amyloid precursor-like protein 1 and 2 (APLP1/2) do not bear the Aβ sequence [17, 27]. Mice that have had their APP or ALPL genes knocked-out are both viable and fertile. However, removing APLP2 and either APP or APLP1 induce death early after birth [27]. These observations suggest that APP and APLP1/2 possess partial functional redundancies, and that Aβ is not essential for survival, though its conservation in the animal kingdom implies positive selection properties during evolution.

Current evidence suggests that, in physiological circumstances, sAPPα and sAPPβ act as neurotrophic factors capable of stimulating both neuronal survival and neuritic growth with each fragment having finely tuned sensitivities in distinct sets of neurons [22, 28, 29].

#### 3.1.2. Amyloid Beta Transport Across Body Compartments

Since Aβ is synthesized by many cells in the body, it may need to be transported to different organs for final elimination when degradation cannot occur locally. To date, the most studied compartment exchange is the crossing of the blood-brain barrier (BBB). Amyloid beta can be transported bi-directionally across the BBB. Roberts and colleagues have reported that direct transport of Aβ monomers, oligomers, and derived diffusible ligands across the BBB represents ~25% of Aβ clearance in humans, while the cerebro-spinal fluid (CSF) absorbs and clears ~25% of the total CNS Aβ [30].

**Figure 2.**
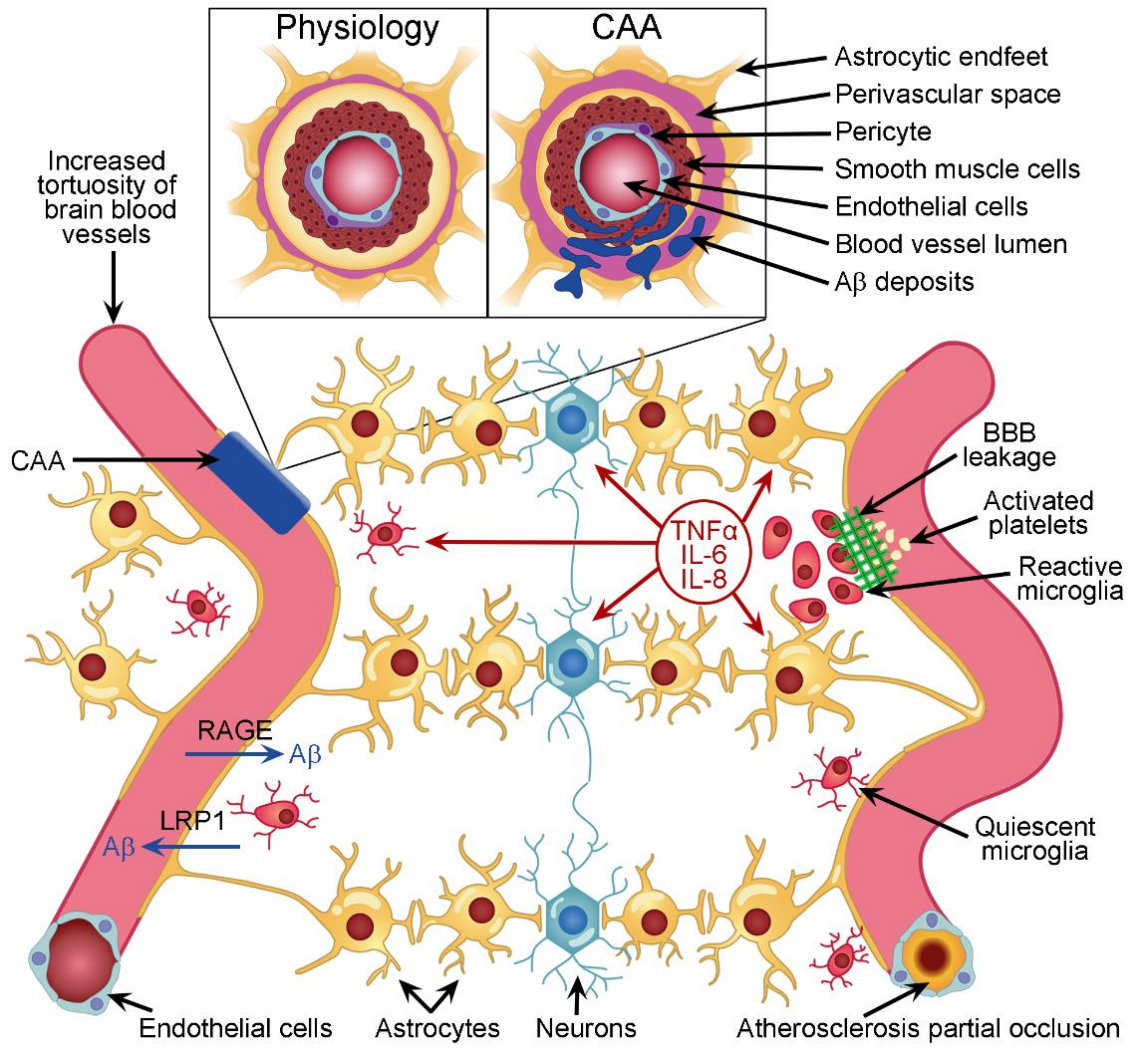
Illustration of some pathophysiological changes that could affect neurovascular units and induce chronic CNS neuronal stress, ultimately initiating AD and its associated neuropathologies. During aging and disease, blood vessels in the brain become more tortuous. This affects the laminar blood flow in the vessels and may provoke the local activation of platelets and/or formation of atherosclerosis. In case of high blood Aβ levels, RAGE receptors are capable of transporting Aβ into the brain parenchyma. Conversely, LRP1 receptors can remove Aβ out of the brain. Both blood and brain Aβ could form multimers that accumulate in the wall of blood vessels to induce CAA (insert), which is often associated with increased perivascular space volume. When the BBB is leaking, some blood material may enter the brain parenchyma (right). The leak is sealed by the aggregation of platelets that initiate clotting. Meanwhile, microglia sense the blood material that has entered the brain parenchyma and initiate an immune response by releasing pro-inflammatory cytokines and chemokines in the milieu to communicate with surrounding cells. If several of these conditions become chronic and overlap over time, then they may initiate permanent neuronal stress leading to the onset of AD.

Amyloid beta produced in the brain parenchyma can efflux out of the brain by binding to cell surface receptors located on the abluminal side of cerebral endothelial cells. A major receptor in this process is lipoprotein receptor-related protein 1 (LRP1; Fig. 2), which comprises a 515 kDa alpha-chain and an 85 kDa beta-chain associated non-covalently, and whose ligands also include ApoE, α2-microglobulin (α2M), and tissue-type plasminogen activator [31]. Similarly, LRP2 is capable of capturing Aβ in presence of apolipoprotein J (ApoJ; also known as clusterin) via endocytosis and intracellular endothelial transport to the luminal side of blood vessels for release into the bloodstream [32]. Another endothelial cell surface receptor is phosphatidylinositol-binding clathrin assembly protein (PICALM), which likely works in conjunction with LRP1 to transport Aβ monomers and oligomers out of the brain [33]. Furthermore, although it is primarily located on the luminal side of the brain endothelium, P-glycoprotein (Pgp) is also capable of transporting Aβ from the brain into the blood [34]. Additional possible transporters have been suggested, but their exact implication in the removal of Aβ outside of the brain remains unclear at the moment.

Opposite to efflux, Aβ influx from the blood into the brain has also been reported. The main transporter identified for this influx is the 35 kDa transmembrane receptor for advanced glycation end products (mRAGE; Fig. 2). In addition to other ligands, mRAGE carries Aβ from the adluminal to the abluminal side of endothelial cells via transcytosis. Soluble forms of RAGE (sRAGE) are also present in the circulation and can capture blood Aβ to transport it to organs capable of catabolizing monomeric and oligomeric Aβ, such as the liver and kidneys [35, 36]. Importantly, although both influx and efflux mechanisms have been described, it remains unclear whether these processes occur readily in physiological conditions, or whether they primarily take place during pathological events, and, if the latter, whether brain Aβ flux is triggered by any pathology described in section D or by a very limited number and type of pathological events.

Other pathways reported to remove Aβ out of the brain involve the direct drainage of CSF into peripheral blood via arachnoid villi and granulations in the walls of major venous sinuses [37]. More recently, a glymphatic system has been described in rodents [38], and this pathway may also occur in humans, although the latter possibility is still debated [39]. In short, the astrocytic endfeet forming the BBB do not directly contact endothelial cells, but rather form a shaft with space between the endfeet and blood vessels where a loose basement membrane allows for the circulation of fluids (Fig. 2 insert). These perivascular spaces have previously been referred to as the Virchow-Robin spaces [40]. CSF from the subarachnoid space can influx inside the brain via the Virchow-Robin spaces around arteries, diffuse into the brain parenchyma where it mixes up with Aβ-containing interstitial fluid (ISF) bathing CNS cells, and efflux out of the brain by diffusing into the Virchow-Robin spaces around veins. The energy to maintain this process appears to come from the pulsatility of the blood flow created by heart beats [38].

Taken together, these studies suggest that (i) Aβ can be transported in and out of the brain via several mechanisms, and that the deficiency in one or more mechanisms could lead to the accumulation of Aβ in the brain parenchyma; and (ii) the local levels of Aβ are tightly regulated not only in the brain, but also in the periphery, suggesting that Aβ plays important physiological roles.

#### 3.1.3. Amyloid Beta Elimination

**Figure 3.**
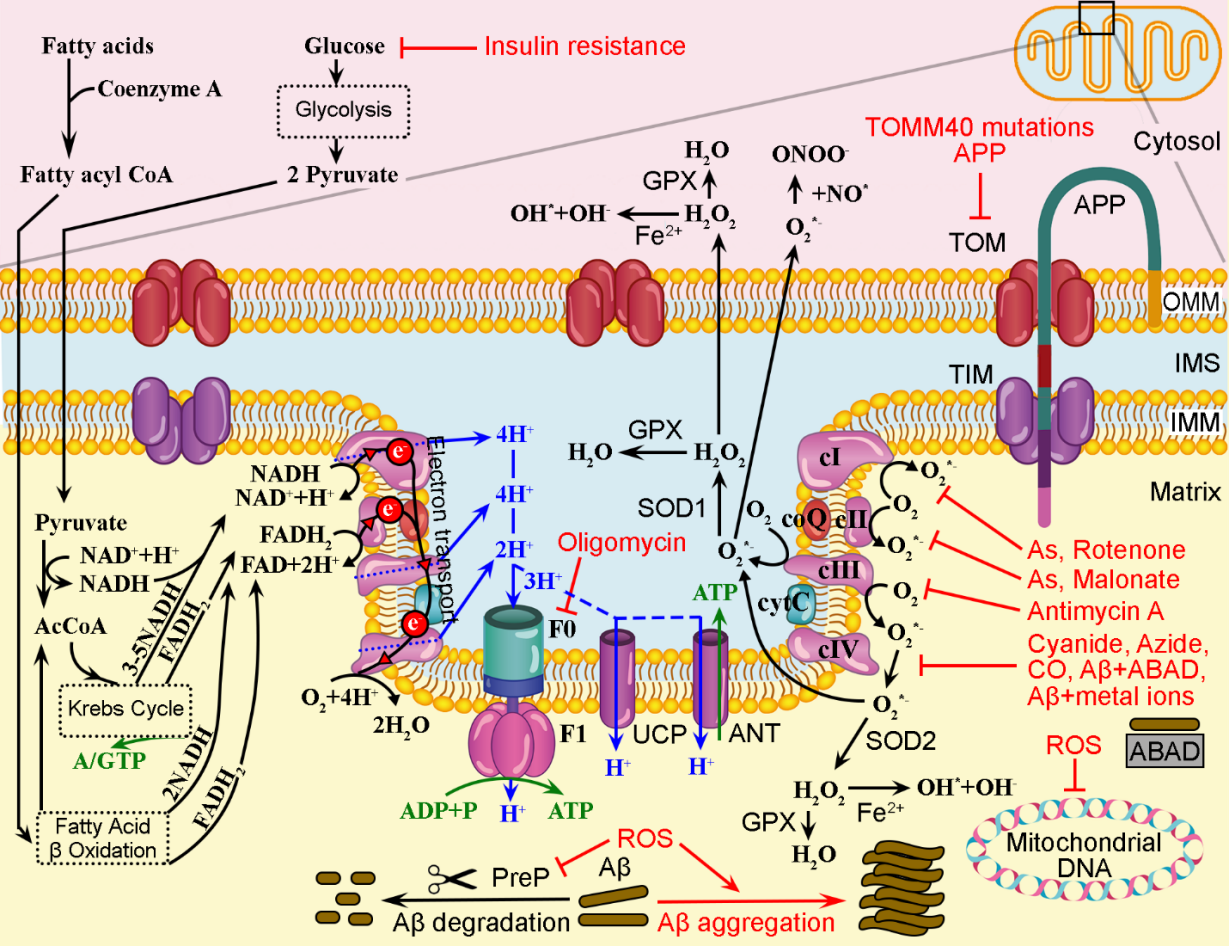
Simplified schematic of the potential mitochondrial pathophysiological challenges in the CNS that may initiate AD. Represented are the main events taking place in mitochondrial cristae formed by the outer and inner mitochondrial membranes (OMM, and IMM). In physiological conditions (left), fatty acids and glucose from the cytosol fuel the Krebs cycle with Acetyl-coenzyme A (AcCoA) to generate reduced nicotinamide adenine dinucleotide (NADH) and reduced flavin adenine dinucleotide (FADH_2_). Oxidation of NADH provides electrons to the first membrane-bound complex (cI) in the mitochondrial respiratory chain, while oxidation of FADH_2_ provides electrons to cII. Electrons are transported to coenzyme Q (coQ), then to cIII, cytochrome C (cyt C), and cIV where they are used to combine oxygen (O_2_) and protons to form H_2_O. When electrons are transferred to cI, cIII, and cIV, the same complexes transport protons from the matrix into the intermembrane space (IMS) to create a proton gradient. Protons return to the matrix by passing through the ATP synthase formed of two subunits named F0 and F1, which generates ATP that can be used for biochemical reactions by cellular enzymes. Alternatively, protons can leak into the matrix via other proteins, such as uncoupling protein (UCP) and ATP exchanger adenine nucleotide translocases (ANT). Proteins produced in the cytosol can enter the IMS via translocase of the outer membrane (TOM) and the mitochondrial matrix via translocase of the inner membrane (TIM) supercomplexes. Low amounts of Aβ may also enter mitochondria, but can be degraded by Presequence protease (PreP). In unfavorable conditions (right), insulin resistance can decrease the transport of glucose into the cytoplasm of neurons, which will affect the electron transport chain and production of ATP. TOMM40 mutations and the presence of APP in the TOM/TIM supercomplexes can impede protein transport into the mitochondrial matrix. Several pollutants and toxins can inhibit the respiratory chain complexes. For example, the insecticide rotenone can inhibit the proper transport of electrons from cI, which will then combine with O_2_ to form superoxide radical anions (O_2_^*-^). The naturally occurring and industrial compounds malonates inhibit cII and induce the formation of O_2_^*-^. Arsenic (As) can inhibit both cI and cII. The toxin Antimycin A produced by *Streptomyces* bacteria can inhibit cIII. Industrial pollutants like cyanide, azide, CO, as well as endogenous Aβ bound to matrix proteins like Aβ binding alcohol dehydrogenase (ABAD, also known as 17β-hydroxysteroid dehydrogenase type 10) or metal ions can inhibit cIV. Oligomycin A, also produced by *Streptomyces* bacteria, is an inhibitor of F0, thus inhibits ATP production. Superoxide radical anions can be catalyzed by superoxide dismutase 1 (SOD1) in the IMS, or SOD2 in the matrix, into O_2_ and hydrogen peroxide (H_2_O_2_). Glutathione peroxidases (GPX) and catalases (not shown) can reduce free H_2_O_2_ to water. When ROS levels increase, anti-oxidant mechanisms become overwhelmed, which may induce the synthesis of RNS. ROS can damage DNA and RNA, as well as inhibit PreP, which will stimulate the aggregation of Aβ inside the mitochondrial matrix.

There are numerous ways for Aβ to be eliminated. In the brain, Aβ monomers and oligomers can be phagocytosed by microglia, and, when necessary, by astrocytes [41]. In the ISF, some soluble enzymes are capable of catabolizing Aβ monomers. These include neprilysin (NEP), insulin degrading enzyme (IDE), and angiotensin converting enzyme (ACE) [31]. Another possibility is that Aβ binds to other extracellular proteins and is transported out of the brain, as explained in the above section. One of these proteins is ApoE, which was shown to enhance the LRP1 transport of Aβ outside of the brain [32]. Intraneuronal Aβ can be degraded by lysosomal autophagy [42]. Furthermore, *in vitro*, Aβ was shown to be imported into the mitochondria through the TOM/TIM complexes. Once inside the mitochondrial matrix, the 110 kDa metalloprotease presequence protease (PreP) is capable of degrading Aβ to reduce the toxic effects of Aβ on mitochondrial functioning [43, 44] (Fig. 3).

In the periphery, Aβ can be degraded in numerous ways (summarized in [31, 45]). First, Aβ can be catabolized by circulating enzymes, such as NEP, IDE, ACE, and plasmin. Several of these enzymes are also present in organs where they are anchored to the plasma membrane, including NEP in the kidney and IDE in the liver [31]. In addition, Aβ can be phagocytosed or endocytosed by leukocytes. Another fashionable way for Aβ to be degraded is by binding circulating erythrocytes to be transported to the liver and spleen where the cells and their Aβ cargo can be eliminated [31]. In addition to cells, Aβ can bind to circulating proteins such as ApoE, ApoJ, α-2M, albumin, sRAGE, and soluble LPR1 (sLRP1). It is estimated that sLRP1 sequesters up to 90% of plasma Aβ [46]. The three major organs clearing Aβ are the liver, kidneys, and spleen. LRP1-bound Aβ can be degraded by hepatocytes, which release Aβ into the bile and thus eliminate Aβ via feces [36]. Radiolabeling experiments have also shown that soluble Aβ can be excreted unprocessed directly in the urine [47].

Given the diverse ways for Aβ to be degraded, one can infer that the dysfunction of one or multiple elimination mechanisms may result in increased levels of Aβ in the entire body, which could lead to the development of chronic disorders and increase neuronal stress in the CNS.

#### 3.1.4. Main Amyloid Beta Physiological Functions

Although Aβ peptides have been implicated in senile plaques found in AD patients since the 1980’s, and despite the fact that its high degree of conservation in the animal kingdom suggests an evolutionary advantage, identifying the physiological functions of Aβ has proven very challenging. This is due in part to the fact that Aβ peptides are very sticky and bind to many proteins and laboratory vessels, making it very difficult to handle and analyze these peptides. Further, APP knock out (KO) mice don’t show any major disturbances at first glance. Only after careful examination of APP KO mice and improving laboratory analytical procedures definite physiological functions for Aβ have been identified [48]. The key point is that most of the physiological activities of Aβ occur when the peptides are expressed at low concentration. This is the case for the regulation of synaptic plasticity, as it was observed that low levels of Aβ enhance long-term potentiation in hippocampal neurons and thus modulate learning and memory [49]. Another physiological role is the scavenging of free metal ions like Al^3+^, Cu^2+^, Fe^3+^, and Zn^2+^, preventing these free metal ions from promoting oxidative reactions. Furthermore, during infections of human cells Aβ is capable of opsonizing several microbial pathogens to facilitate phagocytosis by monocytes, and also possesses bactericidal, fungicidal, and virucidal activities. For example, *in vitro* Aβ42 can neutralize the infection of neuron-glia co-cultures by herpes simplex virus 1 (HSV1) [50]. This has led some investigators to propose that Aβ plaques found in the brain of cognitively normal individuals may actually reflect previous neutralization of CNS microbial infections [48], though this hypothesis is still debated. Moreover, Aβ seems to be involved in homeostatic activities by facilitating clotting after rupture of the endothelium in blood vessels, including along the BBB [51], which would explain, in part, why platelets produce Aβ.

In summary, Aβ is synthesized by many cell types in the body and is finely tuned to exert its physiological roles. This suggests that, if Aβ levels are altered without caution, for example by chronically sequestering or eliminating too much Aβ, then symptoms related to changes in physiological functions may manifest (e.g., abnormal blood clotting). This is precisely what occurred when anti-Aβ immunotherapies were administered to AD patients, which resulted in a high incidence of brain amyloid-related imaging abnormalities with edema (ARIA-E) or microhemorrhage (ARIA-H) [52].

### 3.2. Amyloid Beta is Generated During Neuronal Stress

AD is characterized pathologically by the presence of aggregated Aβ peptides in senile plaques in the brain. However, the missing link in the amyloid cascade hypothesis is the reason why Aβ becomes overexpressed in the first place in non-genetic, sporadic cases of AD. Crucially, during CNS insults Aβ is produced in large quantities by neurons under stress or injury. These observations have led some investigators to suggest that APP and Aβ are actually an adaptive, protective response to insults and part of the immune response in the CNS [15, 16], and can act as a metabolic marker of neuronal stress. Thus, any condition that would reduce oxygenation, the delivery of nutrients, and/or the production of energy in CNS neurons has the potential to lead to neuronal distress and produce increased levels of Aβ. Below we discuss the major alterations that can lead to neuronal stress and were shown to induce transient and/or chronic Aβ overexpression.

#### 3.2.1. Hypoxia and Starvation

Numerous scientific reports have shown that neuronal cell lines and primary neurons in culture overexpress APP and produce large amounts of Aβ when placed in hypoxic conditions [53]. This is seen in pheochromocytoma (PC12) cells [54], mouse/rat primary hippocampal [55] and cortical neurons [56], human induced pluripotent stem cells (iPSCs) differentiated into neurons [57], as well as neurospheroids [58]. Hypoxia occurs when cellular oxygen levels fall in the range 0.5-5%. Under such hypoxic conditions, mitochondria are not fueled with enough oxygen and acetyl-CoA, causing higher production of intermediate ROS and nitric oxide (NO) than normal (Fig. 3), which induces intracellular stress [59]. Very interestingly, when ROS are produced in higher amounts in mitochondria, they inhibit PreP, thus Aβ degradation. This leads to the accumulation of Aβ inside the mitochondrial matrix, which in turn inhibits Complex IV of the electron transport chain, creating a vicious circle responsible for increasing the generation of ROS and RNS [60, 61] (Fig. 3). Although the exact reason why Aβ enters mitochondria in physiological conditions is unclear, one could speculate that, because of its strong binding properties, mitochondrial Aβ may bind to free metal ions and damaged proteins in order to prevent the catastrophic lysis of mitochondria that could happen in case of excessive ROS production (Fig. 3). In this scenario, Aβ would act as an anti-stress agent that would preserve the integrity of mitochondria and, more globally, cells. In transgenic APP23 mice overexpressing human APP with the Swedish (swe) double mutation 670/671 (KM→NL), hypoxia 16 hours/day for 1 month significantly increased Aβ loads in the brain [62]. Similar results were found in APP^swe^/PS1^dE9^ mice placed in hypoxic conditions for 24 hours [63], and in male 3xTg-AD mice undergoing a chronic intermittent hypoxia/reoxygenation paradigm for eight weeks [64]. These results demonstrate that acute hypoxia is sufficient to induce Aβ expression in the brain. If the extracellular milieu improves, then neurons can be rescued. However, if the milieu doesn’t improve, or worsens, leading to a chronic stress situation, then neurons may activate apoptotic pathways, as demonstrated by the expression and activation of caspase-3 and -9 in primary cultures of mouse cortical neurons under hypoxic conditions [56]. Very interestingly, one study has shown that knocking out either APP or BACE1 in mice undergoing a transient, bilateral occlusion of the common carotid arteries for 12 minutes results in higher death rates compared to wild-type mice [65]. However, additional experimentation is necessary to demonstrate whether the higher mortality rate in KO mice is due to the lack of Aβ, as this preliminary study did not address this specific mechanism.

In addition to hypoxia, another way for neurons to be starved is by lacking nutrients, for example by the thickening of blood vessel walls, which results in impaired diffusion and transport of nutrients to tissues. Under nutritional stress, neurons stop operating optimally, nutrients are not fueled to the Krebs and fatty acid β-oxidation cycles to produce acetyl-CoA that is used by mitochondrial respiratory Complex proteins I-IV to produce adenosine triphosphate (ATP; Fig. 3), ultimately resulting in low intraneuronal energy stock and increased ROS production, which alters the electrical activity of neurons. If the condition persists, neurons can experience glutamate excitotoxicity and/or apoptosis [66]. Beyond neurons, there is evidence that primary umbilical vein endothelial cells (HUVEC) produce Aβ42 under nutrient starvation [67].

#### 3.2.2. Structural Integrity Challenges

Changes in structural integrity of CNS neurons and supporting cells include stretching or severing of cells and neurites due to shear forces and foreign objects. Such application of mechanical forces, for example, by dissecting out primary neurons for *in vitro* cultures, leads to a stress response in CNS neurons and their neurites, including an increase in expression of APP and Aβ [68]. Similar results are found in animal models of traumatic brain injury (TBI), including in wild-type mice, which display a transient (a few days) deposition of Aβ in the vicinity of the site of injury that resorbs naturally [69]. Very interestingly, TBI carried out on the transgenic AD mouse model PDAPP co-expressing human ApoE3 or ApoE4 (hApoE3/4) have shown that hApoE4 precipitates the deposition of Aβ in the brain [70]. This is likely due to the fact that hApoE4 is less efficient at clearing Aβ out of the brain than hApoE2 and hApoE3 in PDAPP mice [71]. Therefore, careful analysis of the literature indicates that Aβ is transiently synthesized by neurons during acute traumatic events that affect their structural integrity. However, in traumatic conditions outside of chronic neurodegenerative disorders, transient Aβ overexpression seems to act like a protective/survival mechanism to prevent neuronal apoptosis and necrosis.

#### 3.2.3. Inflammation and Pathogens Challenges

As indicated in section C.1.d. above, it was observed that human H4 neuroglioma cells produce Aβ upon exposure to HSV1 [50] and other viruses, bacteria and fungi. In this scenario, Aβ is protecting CNS cells from infection and death. Aside from microbial infections, other causes that can induce an inflammatory response include structural integrity challenges or peripheral inflammation transmitting into the CNS via the choroid plexus [72]. Inflammation is sensed in the CNS milieu mainly by microglia and astrocytes, which communicate the information to neurons [73] (Fig. 2). In turn, neurons and glial cells express inflammatory molecules, and Aβ [74]. In chronic inflammation situations, neurons and surrounding glial cells may continuously express Aβ, which then aggregates into toxic forms, i.e., oligomers and protofibrils. Amyloid beta-induced neurotoxicity has, in part, been attributed to the oxidant potential of Aβ. For example, inoculating Aβ-containing senile plaque extracts in the brain [75, 76] or peritonea [77] is sufficient to accelerate Aβ deposition and neuronal death in APP transgenic mice. This process seems to involve the production of ROS through metal ion reduction [78].

To summarize this section, Aβ appears to have several physiological roles in the body. Physiological functions happen primarily at low levels of Aβ, and the levels seem to increase when there is a microbe to manage or insults to resolve. Overexpression of Aβ by CNS neurons and glial cells can emanate from multiple stressors. Critically, only in chronic conditions and at concentrations high enough to create protofibrils does Aβ become toxic and amplify the inflammatory pathological processes already initiated in the CNS. This suggests that transient high levels of Aβ alone are likely not sufficient to induce the cascade of events leading to AD and related dementia. Instead, we propose that the primary etiological driver of AD is the convergence of multiple and overlapping chronic central and peripheral pathologies that impact negatively the composition and metabolism of the CNS milieu (Fig. 1), thereby creating a deleterious environment that induces persistent neuronal stress and Aβ overexpression by several CNS cell types. Consequently, if not returned to homeostasis, continual neuronal stress will promote and exacerbate diverse neuropathologies (e.g. phospho-tau and higher levels of Aβ42) in a vicious cycle that ultimately progresses to neurodegeneration and associated cognitive and neuropsychiatric symptoms typical of AD, as outlined in the current hypothetical model of AD biomarkers evolution [12].

## 4. Human Chronic Pathologies Leading to Neuronal Stress

AD pathological hallmarks are the presence of Aβ senile plaques and NFT in the brain of patients. In the previous section, we have summarized the current knowledge about Aβ metabolism and physiology that support our innovative theory on AD etiology. In the next sections we will specify several chronic human pathologies that could induce neuronal stress in the CNS, thus be determinants of AD (summarized in Table 1). We will not report on genetic mutations leading to ~5% of AD cases (familial AD; see introduction) as these were discussed in a previous review [79], but rather focus our attention on conditions that could explain the remaining 95% of AD cases (sporadic AD). We want to emphasize that a single chronic condition is unlikely to induce AD. Instead, the overlap of multiple chronic disorders described below is the most probable scenario (Fig. 1). While the vascular hypothesis is the most comprehensive theory to date, it is still incomplete. Thus, we propose that diseases beyond cardio-vascular dysfunctions are likely to be involved in the etiology of AD.

**Table 1:** Examples of human conditions inducing different types of stress within the CNS. See text for details.

Human Chronic Conditions	Induced CNS Stressor
CNS Alterations	
BBB Dysfunction	Inflammation
Repeated Traumatic Brain Injury	Inflammation
Cerebral Amyloid Angiopathy (CAA)	Hypoxia and Starvation
Hormonal Imbalance	Starvation
Sleep Disturbance	Hypoxia and Inflammation
Metabolic Disorders	
Chronic Liver Disease	Starvation and Inflammation and Increased Aβ
Metabolic Syndrome	Inflammation
Diabetes	Starvation and Inflammation
Obesity and Unbalanced Diets	Inflammation and Oxidative Stress
Changes in Blood Flow and Composition	
Cardiovascular Disease	Hypoxia and Starvation
Abnormal Blood Pressure	Hypoxia and Starvation
Atherosclerosis	Hypoxia and Starvation
Vascular Inflammation	Inflammation
Low and High Hemoglobin Levels	Hypoxia
Inflammation and Toxic Agents	
Microbial Infections	Inflammation
Systemic Inflammation	Inflammation
MItochondrial Dysfunction	Oxidative Stress
ROS / RNS	Oxidative Stress
Smoking	Hypoxia and Inflammation and Oxidative Stress
Alcohol	Inflammation (BBB leakage)
Environmental Toxins and Metals	Inflammation and Oxidative Stress
Autoimmune Diseases	Inflammation and Oxidative Stress
Other Conditions	
Chronic lung disease	Hypoxia
Chronic Kidney Disease	Inflammation and Increased Aβ
Aging	Inflammation and Oxidative Stress

### 4.1. Mitochondrial Dysfunction

Mitochondria are the powerhouse of cells. They use oxygen to oxidize nutrients via four respiratory chain complexes that manipulate electrons and protons to ultimately generate ATP [26] (Fig. 3). Therefore, any condition that affects mitochondrial energy production has the potential to reduce the energy available to cellular physiological processes, which may induce apoptosis. There are two types of human mitochondrial conditions that are linked to AD. Firstly, mutations in key mitochondrial genes affecting the transport of proteins or nutrients would reduce the amount of fuel for the Krebs and fatty acid β-oxidation cycles, thus lower the quantity of ATP generated by mitochondria. A chronic decrease in ATP in CNS neurons will lead to the production of ROS and RNS, thus Aβ [80] in an attempt to guide inefficient mitochondria to mitophagy. For example, polymorphisms in the *TOMM40* gene are associated with an earlier onset and increased risk of developing late-onset AD [26]. An intriguing and not yet fully understood mechanism is the presence of APP in the TOM pore formed by 40 kDa TOM40 proteins, which seems to alter the proper transport of proteins inside mitochondria during pathological events [26] (Fig. 3). This was observed *in vitro*, in rodents, and in human brain tissues [81, 82]. Furthermore, *in vitro* studies have shown that γ-secretase is present in the outer membrane of mitochondria and can cleave APP bound to TOM40, though it appeared that the mitochondrial cleavage of APP uses principally the non-amyloidogenic pathway as BACE1 was not identified in purified mitochondria [82]. Nonetheless, the current hypothesis is that agents capable of altering the physiological interactions between TOM40 and cytosolic proteins, such as APP, would disturb the importation of materials inside mitochondria and disrupt the regulation of the redox potential [81]. However, additional work is needed to fully understand this unusual mechanism and determine whether it takes place in the human brain outside of pathological conditions.

The other mitochondrial condition linked to AD is the decoupling of electron and proton generation by high levels of metal ions, toxins, and chemicals. The most common environmental toxins generated from industrial and agricultural activities are pesticides, smoke, mycotoxins, polychlorinated biphenyls (PCBs), and arsenic [83], plus environmental pollutants including heavy metals [84]. Toxins can inhibit the mitochondrial respiratory chain complexes, and free metal ions can increase the production of ROS, which have been directly linked to neuronal toxicity, synthesis of Aβ (Fig. 3), and an increased risk of developing AD [85]. A famous case illustrating the aforementioned scenario involves identical twin brothers with the one working in a pesticide plant developing AD, while the brother working in a different environment devoid of toxic chemicals did not suffer from AD [86]. Importantly, ROS generated in the mitochondrial matrix can inhibit PreP, which would result in the accumulation of Aβ inside mitochondria (Fig. 3). In turn, high levels of Aβ are capable of decoupling Complex IV, thereby forming a vicious circle perpetually generating ROS and increasing intramitochondrial Aβ levels [43]. Although it seems unlikely that alteration of mitochondrial transport, or the chronic exposure to toxins and chemicals alone are sufficient to induce AD, such environmental challenges may combine with other chronic pathologies to stimulate to the initiation of AD.

### 4.2. Oxidative Stress

Importantly, the majority of all mitochondrial functionality is altered in AD [87], including ROS and RNS. In 1999, Markesbery and Carney elegantly summarized the knowledge about oxidative pathologies in the brain of post-mortem AD patients [88]. Shortly later, Nunomura et al. reported that oxidative damage was the greatest early in the disease, then was reduced with disease progression, which was associated with increased brain Aβ loads [89]. These observations led to the hypothesis that oxidative stress plays a critical role in the disease pathogenesis [87, 89, 90]. In short, biochemically, ROS and RNS are derived from the incomplete reduction of oxygen in mitochondria and peroxisomes [91]. ROS and RNS synthesis and clearance are intricately linked [92] (Fig. 3). Physiologically, they are produced at low levels and neutralized by non-enzymatic and enzymatic antioxidants (e.g. glutathione, flavonoids, superoxide dismutase, and catalases). During aging, mitochondria and other organelles increasingly leak ROS. The rise in ROS levels also stems from Aβ-related microglial activation, inflammation, and binding of redox metals [93]. Conversely, both the activity of antioxidant enzymes and the intake of non-enzymatic antioxidants decrease, which exacerbates the overall increase in ROS and RNS in the body and brain [94]. High levels of ROS induce lipid and cholesterol peroxidation (e.g., disruption of membrane integrity), protein and glycoprotein oxidation (e.g., LPR1 oxidation which impairs Aβ clearance), and DNA and RNA oxidation (including mitochondrial DNA), all of which perturb homeostasis [95]. In addition, high levels of metal ions-bound Aβ in mitochondria are associated with high levels of ROS [95]. Interestingly, markers of oxidative and nitrosative stress have been reported in blood, CSF and urine in early and late stages of AD [96], indicating they are a constant presence during AD progression, which could be exploited as a potential biomarker to follow disease trajectory [97, 98].

### 4.3. Conditions Altering CNS Structural Integrity and Homeostasis

As mentioned in section C.2.b., TBI and the accidental introduction of foreign objects in the CNS can induce cellular and axonal damage. It is generally accepted that the more severe the TBI, the more inflammation will take place at the site of injury. In humans, the levels of Aβ protofibrils were found to be increased in brain tissues from patients who have experienced TBI within one week of biopsy [99]. Furthermore, Aβ42 levels in the ventricular CSF were increased more than 1,000% within 5-6 days in patients that suffered a severe TBI and remained elevated during days 7-11 [100]. To note, each patient had a different individual timeline for changes in Aβ42 levels, likely reflecting the type and force of the endured TBI, and the investigators did not report any data after day 11. The increase in brain amyloid burden after TBI was also observed by amyloid positron emission tomography (PET) in a small sample of patients. The authors reported that amyloid PET distribution overlapped with, but was distinct from, that of AD [101]. Another study has confirmed that transient Aβ deposits may be observed by amyloid PET imaging after TBI [102]. In another neurodegenerative disorder, i.e., secondary progressive multiple sclerosis, the axonal pathology associated with demyelination leads to increased levels of Aβ in white matter tracts, which is currently been evaluated as a biomarker of neurodegeneration and demyelination via amyloid PET [69, 103]. Furthermore, Aβ is also found in the brain of patients suffering Lewy body dementia (LBD) and is detectable by amyloid PET [104, 105]. Based on our clinical practice and the analysis of numerous pathological reports, we advance the idea that the overexpression of Aβ in LBD and Parkinson’s disease is due to the persistent stressing of CNS neurons that occurs after the alpha-synuclein pathology alters homeostasis in the CNS. This interpretation is in agreement with the theory proposed by Hansen et al. [106].

Similarly, gliosis and progressive axonal degeneration are regularly noted in early stages of AD and linked to Aβ and tau accumulation [107]. In addition to Aβ and tau pathologies, the BBB may also be altered, thereby perturbing cerebral blood flow, which may contribute to brain ischemia, neuronal degeneration, and synaptic dysfunction [108] (Fig. 2). From population studies, it is estimated that 10-20% of individuals suffering a single severe TBI or repeated mild TBI will develop dementia within the following 5-15 years [109, 110]. A relevant illustration of these findings is provided in a recent report linking a high incidence of traumatic encephalopathy and dementia in a sample of retired soccer players [111]. Such observation raises the key question of whether athletes in high-impact sports should be prescribed prophylactic medications to mitigate the effects of repeated mild concussions during their career.

A lesser-known issue than TBI is the partial demyelination of white matter tracts in menopausal women. This phenomenon seems to be associated with brain starvation after estrogen stops being produced in the periphery. Brain cells then switch from using glucose to lipid-derived ketone products as fuel. The lipids used in this process come largely from myelin sheaths. Since altered myelin negatively impacts axonal conduction, which is linked to lower cognitive abilities, and because starvation leads to neuronal stress, it is possible that the demyelination process may lead to AD if diet and/or hormonal adjustments are not deployed early after menopause [112]. This post-menopausal demyelination process could partly explain why women are more prone to develop AD than men.

### 4.4. Cardiovascular and Blood Diseases

The link between cardiovascular diseases and AD is the basis for the vascular dysfunction hypothesis [11]. Here, we summarize the main information potentially linking cardiovascular disease to Aβ overexpression; we refer readers to the review by Klohs for full details about the vascular dysfunction hypothesis [11]. Firstly, one can anticipate that any disease affecting heart functioning will likely alter blood flow and the delivery of oxygen and nutrients to the brain. In support of this idea, it was shown in rats that the reduction in heart performance after induced infarct was directly linked to an increase in Aβ levels in the brain [113]. To note, such study is difficult to carry out in humans for ethical reasons.

Secondly, conditions affecting the elastic properties of blood vessels would affect brain perfusion. Those include hypertension, hypotension, hypercholesterolemia, atherosclerosis, chronic peripheral inflammation, increased peripheral ROS levels, and diabetes. These chronic vascular insults can stimulate RAGE activity, and thus increase the influx of Aβ from the blood into the brain [11]. The main risk factor for changes in blood vessel biophysical properties is aging. Indeed, during aging, blood vessels naturally become stiffer. This changes the laminar flow of blood to a more pulsatile rhythm, which impairs the absorption of nutrients across the BBB and has been strongly associated with cognitive impairment [114]. Aging is also associated with vascular inflammation. The process is partly guided by the exposure of endothelial cells to increased levels of circulating Aβ from various sources, which promotes the release of a myriad of inflammatory mediators such as IL-1β, IL-6 and prostaglandins [11], and alters the elasticity of blood vessels. Another anomaly developing with age is a dynamic change in blood vessel density and increased tortuosity, which results in brain hypoperfusion. The increased tortuosity augments shear stress on the walls of blood vessels, which activates platelets and endothelial cells, resulting in the formation of fibrin and the adhesion of leukocytes that partly constrains blood flow in the brain [11] (Fig. 2). This phenomenon can occur in parallel to an increased risk of atherosclerosis (Fig. 2), which can limit further the volume of blood delivered to the brain. Such atherosclerosis was observed in the arteries forming the circle of Willis at the base of the brain, with an overall increased rate of occlusion of these arteries in AD vs. non-AD post-mortem samples [115]. This observation was confirmed in a small sample of living subjects using two-dimensional phase-contrast magnetic resonance imaging, which revealed a 20% decrease in cerebral blood flow in AD subjects compared to age-matched controls [116]. As explained in several parts of our manuscript, limited blood flow due to blood vessel occlusion has strong potential to induce hypoxia and starvation, thus creating a stressful environment for CNS neurons that is possibly associated with an overexpression of Aβ.

Thirdly, AD is often described as a disease with transient BBB leakages. For example, neuropathological studies have found that plasma proteins such as fibrinogen, thrombin, albumin, immunoglobulin G and red blood cell degradation products extravasated into the brain and CSF of AD patients [117]. This is suspected to generate central inflammation and neuronal stress [118, 119]. BBB impairment was more severe in *APOE* ε4 carriers than non-carriers [120]. Animal studies have shown BBB impairment in transgenic mouse models of both amyloidosis and tauopathy [119, 121], and the loss of pericytes may also participate in BBB damage [122]. Moreover, studies have shown that BBB impairment occurs early during AD, before the onset of Aβ deposition, cerebrovascular amyloid angiopathy (CAA) and cognitive impairment [123], suggesting that BBB impairment could induce neuronal stress prior to the formation of senile plaques.

Fourth, CAA causes hemorrhagic stroke and was shown to induce neurodegeneration [124]. CAA is characterized by Aβ deposits in the wall of small arteries, arterioles and capillaries [125] (Fig. 2, insert). CAA was observed in approximately 90% of AD patients and in 50% of the population over the age of 90 years [126]. Carriers of the *ApoE* ε4 allele have more severe CAA than non-carriers [127]. Both intracerebral and circulating Aβ are suspected to contribute to CAA, thus implying that vascular dysfunction may be an integral part of AD etiology and pathophysiology in a bidirectional manner [11]. Compellingly, some investigators have observed that, during CAA, Aβ may first saturate the blood vessel wall and then diffuse in the parenchyma where it forms perivascular senile plaques [128]. Crucially, cerebral hypoperfusion accelerates CAA and promotes cortical microinfarcts [129]. In CAA, Aβ can also accumulate in blood vessel smooth muscle cells [130] and affect the physiology of pericytes, which then induce the constriction of capillaries [131]. Furthermore, platelets show an increased adhesion in the blood vessels of mice with CAA and accumulate at vascular wall sites near Aβ deposits [132]. Platelets were found to be more activated in AD patients [133], which correlated with the rate of cognitive decline [134]. In summary, CAA is likely involved in the leakage of the BBB [135], thus also participates in the development of brain inflammation and neuronal stress.

Fifth, the glymphatic system is suspected to use cardiac pulsatility and blood vessel elasticity as its driving force [38]. If this hypothesis is correct, then any change in the properties of the cardiovascular system and blood flow properties would also impede the glymphatic system, and thus reduce the removal of Aβ out of the brain parenchyma.

Sixth, the excess or diminution of hemoglobin levels have been linked to the doubling of the risk of developing dementia. The first quantification of these effects was reported in 2009 by the group of Dr. Bennett using data from patients enrolled in the Rush Memory and Aging Project. They concluded that both low and high hemoglobin levels in the elderly were associated with a lower level of cognitive function [136]. Further correlational analysis of their cohort revealed that hemoglobin level abnormalities were associated with an increased risk for developing AD and a more rapid cognitive decline [137]. In the past decade, additional reports from other study sites have provided similar observations [138-141]. In our own clinical practice, we have encountered several cases of anemia among our MCI and AD patients. While some authors have suggested that intraneuronal levels of hemoglobin differ at distinct disease stages, thus participating in neuronal stress, and that therapeutics like erythropoietin could help modulate extra- and intraneuronal levels of hemoglobin [142], more work is needed to better understand the molecular links between hemoglobin levels and cognitive decline.

### 4.5. Systemic Inflammation Stimulating Central Inflammation

Systemic inflammation is a major pathology in the metabolic syndrome [143]. Peripheral inflammatory events may propagate into the CNS by different pathways and induce neuronal stress. As indicated in section C.2.c., peripheral inflammation mediators like cytokines and chemokines can enter the CNS via the choroid plexus and diffuse into the brain parenchyma [72]. Another possibility is the entry of peripheral microbes into the brain via transcytosis directly across the BBB or by travelling inside leukocytes, or via retrograde transport from peripheral and olfactory nerves [144]. Interestingly, chronic inflammation due to metabolic disorders can also propagate into the CNS by the entrance of inflammatory molecules in the brainstem area, which may affect cholinergic nuclei (e.g. laterodorsal pontine tegmentum and pedunculopontine tegmental nucleus) that have efferences to the cortex and basal forebrain cholinergic nuclei (e.g. medial septal nucleus, diagonal band of Broca, and nucleus basalis magnocellularis) [145]. There is also evidence that peripheral inflammation due to obesity precipitates brain inflammation [146]. Taken together, these findings suggest that peripheral and central inflammation are interrelated and are linked to cognitive impairment and cholinergic dysfunction taking place in patients suffering from obesity and other metabolic disorders [145].

### 4.6. Chronic Liver Diseases

The liver is a major organ for glucose and lipid metabolism, and detoxification [147]. In metabolic diseases, such as obesity, diabetes, and cirrhosis, the expression of metabolic enzymes decreases [148]. Importantly for AD, hepatocytes produce and release sLRP1 in the bloodstream. Hepatocytes also clear Aβ from the blood by (i) capturing the peptides and releasing them in the bile; (ii) via degradation by IDE, which is highly expressed in hepatocytes; and (iii) by endocytosis when Aβ is bound to sLRP1, ApoE, and cholesterol [149]. Therefore, any chronic liver disease increases the risk of stressing CNS neurons by decreasing the peripheral hydrolysis of Aβ, due to the decreasing levels of IDE and LRP1, and participating in insulin resistance [31]. For example, hepatitis B and C viruses, as well as high alcohol consumption can induce cirrhosis. Hepatitis C infections have been found to be associated with dementia in a large population-based cohort in Taiwan [150]. Cirrhosis, and particularly hepatitis B-induced cirrhosis, was associated with increased plasma levels of Aβ, interleukin-1β (IL-1β), and IL-6 in a group of 46 Chinese patients compared to non-disease controls [151]. Moreover, non-alcoholic fatty liver disease (NAFLD) is the leading cause of chronic liver disease worldwide and increasing in prevalence. If not corrected, the condition can progress to hepatitis. NAFLD often occurs in tandem with obesity, type 2 diabetes, and the metabolic syndrome, and is associated with hepatic insulin resistance. NAFLD develops with increased intake of glucose, fructose (e.g. high fructose corn syrup [152]), saturated fat, and high consumption of medications [153, 154]. In a recent study, serum-based markers of chronic liver disease (e.g. total bilirubin, albumin, alkaline phosphatase, alanine aminotransferase and aspartate aminotransferase) were found to correlate with decreased cognitive performance, increased brain amyloid loads, higher CSF tau levels, greater brain atrophy, and reduced brain glucose metabolism in AD subjects from the Alzheimer's Disease Neuroimaging Initiative (ADNI) cohort [155], suggesting that liver dysfunction may participate in the development of AD. However, the study failed to conclude whether liver dysfunction caused AD or was just a consequence of AD. Nonetheless, alcoholism has recently been added to the list of risk factors for AD by a group of experts [1]. NAFLD can be diagnosed by ultrasound. However, there are no studies to date that have investigated the possible correlation between NAFLD and AD.

### 4.7. Chronic Kidney Diseases

The kidney is another pathway for the peripheral elimination of Aβ. Indeed, ^125^I radiolabeled Aβ40 injected into wild type mice distributed mostly in the liver, but also in the kidney where it was filtered unchanged into urine [47]. Very importantly, chronic kidney diseases (CKD) are often associated with cognitive decline and AD, and high levels of Aβ are repeatedly observed in the bloodstream of CDK patients (summarized in [156]). Although the exact mechanisms need to be investigated further, it has been proposed that the link between CDK and AD may involve vascular dysfunction, chronic inflammation, and oxidative stress [156].

### 4.8. Diabetes

Diabetes shares several pathological features with other culprits described in the present essay. Like obesity, diabetes is associated with chronic peripheral inflammation [157]. The hyperglycemia encountered in diabetes induces dyslipidemia, vascular damage, and oxidative stress, which could result in nephropathy and cardiovascular disease [158]. As explained above, all these chronic conditions can induce neuronal stress in the CNS. Interestingly, type 2 diabetes and AD share several pathologies, including insulin resistance (see below), dysregulation of glucose and insulin signaling [159], Aβ deposition [160] and cognitive impairment [161], and mitochondrial dysfunction [162], which is the basis for some researchers to call AD “type 3 diabetes” [163]. Furthermore, several epidemiological studies have demonstrated a higher risk of AD in patients suffering diabetes [160, 164].

It is estimated that 20% of the daily energy used by the human body is utilized by the brain [165]. CNS neurons consume a large amount of glucose to produce the energy they need to maintain homeostasis and assume their functions. Plasma membrane glucose transporters are mainly activated by insulin. Thus, insulin is a key hormone for neuroprotection and neurotrophism, including protection against oxidative stress and Aβ oligomer toxicity. Notably, decreased brain insulin levels and insulin receptor signaling are associated with impaired cognitive function and neurodegenerative diseases [166]. Insulin resistance is created by chronic exposure to insulin or exposure to high levels of insulin, which desensitizes the receptor, resulting in the blocking of the insulin response [167]. A reduction in glucose transporter activity means neuronal starvation, bioenergetic failure, and increased production of ROS by mitochondria, which, as indicated earlier, are all causes for Aβ synthesis by CNS neurons under stress [168]. Insulin resistance induces cerebral glucose hypometabolism, which is highly indicative of impaired brain circuits and AD. This is demonstrated by the hypometabolism identified in many brain regions when carrying out fluorodeoxyglucose positron emission tomography (FDG-PET) imaging in AD patients [169]. Interestingly, some authors have proposed to investigate the concept of brain energy rescue [170], and pilot studies have shown that glucose hypometabolism might be compensated by increasing ketones as fuel supply [171]. However, the veracity of such hypothesis and determining the exact effects of ketones on neuronal stress, or its prevention, need to be tested in larger studies before this type of intervention can be adopted in clinical settings to treat AD. Another possible therapeutic approach would be to use diabetes drugs that regulate glucose intake and insulin signaling (summarized in [163, 172]). Of particular interest at present are glucagon-like peptide-1 receptor agonists (GLP-1 RA). One pilot trial showed an improvement in FDG PET imaging after 6 months of treatment with a GLP-1 RA agent, though no changes in amyloid loads were detected vs. placebo [173]. Additional studies are underway to assess the therapeutic potential of GLP-1 RA in AD.

### 4.9. Obesity and Unbalanced Diets

Obesity is often part of the metabolic syndrome, and thus associated with chronic systemic inflammation and diabetes [157]. Prototypical pathologies of obesity include hyperlipidemia, hypercholesterolemia, insulin resistance, oxidative stress, NAFLD and vascular damage [174], all capable of inducing neuronal stress in the CNS. Similar pathologies are observed in individuals consuming unbalanced meals with few fruits and vegetables, but high in proteins, fast released carbohydrates, and saturated lipids. Such rich diets affect mitochondria, causing them to produce more ROS, thus increasing neuronal stress in the CNS if these dietary habits are not corrected. In addition, obesity and unbalanced diets are often linked to chronic stress, which disrupts the hypothalamus-pituitary-adrenal axis and circadian rhythms [175], which may affect sleep.

### 4.10. Sleep Disturbance

A relatively recent field of study is the role of sleep disturbance in the development and progression of AD. However, the complex interplays between sleep and AD are not fully understood to date [176, 177]. Several processes are at play with sleep disturbance. Firstly, chronic stress and night shifts may affect sleep quality. A single night of sleep deprivation or prolonged wakefulness was shown to interfere with the physiological morning decrease in Aβ42 levels in both healthy elderly subjects and patients with AD. This result made the investigators hypothesize that chronic sleep deprivation increases cerebral Aβ42 levels, which in turn elevates the risk of AD [178]. Secondly, it has been reported that sleep apnea increases brain amyloid loads [179]. This process can be directly linked to a decrease in oxygenation during sleep, which results in neuronal stress, in a similar manner as any chronic lung disease. Thirdly, sleep is a period of the day during which waste is removed out the brain. Recent reports suggest that waste removal may be guided by the glymphatic system [180]. As indicated in section C.1.b., it is suspected that the glympathic system participates in Aβ removal out of the brain. Thus, if sleep disturbance affects the proper functioning of the glympathic system, Aβ may accumulate in the brain of individuals who experience sleep loss.

### 4.11. Smoking and Alcohol

Smokers have a higher risk of developing dementia than non-smokers [181]. While the exact mechanisms underlying this phenomenon remain elusive, one can hypothesize that smoking reduces oxygen levels in the blood, induces hypercapnia (i.e., elevated levels of carbon dioxide in the bloodstream), raises the levels of carbon monoxide in the blood, which competes with oxygen to bind hemoglobin and generates ROS (Fig. 3), and releases many toxins into the bloodstream that will have to be processed by the liver. For example, a study has identified that arsenic, cadmium, chromium, nickel, and lead are present in cigarettes [182], and follow-up studies have reported that smokers had higher blood concentrations of arsenic, aluminum, nickel, and lead than non-smokers [183, 184]. This indicates that the use of tobacco products likely increases systemic oxidative stress. Furthermore, cadmium was found to be elevated in the blood of Korean women exposed to secondhand smoke during their daily activities [185], implying that passive exposure to tobacco smoke is sufficient to increase blood levels of some toxic metal ions. The toxic effects of tobacco product usage stimulates hypoxia, liver detoxification processes, oxidative stress, and vascular damage [186], all of which can induce chronic neuronal stress in the CNS and perhaps increase the risk of developing AD [187]. Very importantly, epidemiological studies have linked the concomitant heavy consumption of alcohol and smoking with an increased risk of developing chronic liver diseases [188]. Given the potential link between chronic liver disease and neuronal stress (see section D.6.), the combination of high consumption of alcohol and smoking is a strong risk factor for developing AD. Furthermore, *in vitro* and animal experiments have shown that the chronic consumption of alcohol makes the BBB leaky by impairing intercellular tight junctions [189, 190]. Although the BBB leakiness in itself may not be sufficient to develop AD, this pathology may combine with other conditions explained above to escalate neuronal stress in the CNS.

## 5. Risk Assessment and Possible Prevention or Reversion of Alzheimer’s Disease

The main risk factor associated with AD is age. One could argue that the medical improvements in the past century have resulted in increased life expectancy, which also increases the risk of suffering age-associated chronic pathologies like those described in section D. A recent large epidemiological study on a sample of 8,456 Taiwanese individuals demonstrated that comorbidities associated with MCI and dementia included cirrhosis, cerebrovascular disease, asthma and diabetes mellitus [191], though no causation effect was proposed. Furthermore, it has been reported that deaths due to cardiovascular disease and stroke have been reduced between 2000 and 2018 [192]. Thus, two key questions emerging from our theory of multipathology convergence to chronic neuronal stress are: (i) is the decrease in cardiovascular disease death, but an increase in the number of older patients living with chronic impaired cardiovascular functions linked to a higher incidence of AD? and (ii) can we use this new theory to improve clinical care and to better understand the heterogenous progression of AD in patients?

In recent years, the group of Dr. Kivipelto has studied the risk of developing dementia using the non-invasive composite Cardiovascular Risk Factors, Aging, and Incidence of Dementia (CAIDE) score [193]. The CAIDE risk score utilizes age, years of education, sex, systolic blood pressure, total cholesterol, and physical activity to determine an individual's likelihood of developing dementia within 20 years. While it provides a decent estimate of the risk of developing dementia, the CAIDE score could be improved significantly by adding additional medical history parameters described in the present essay such as history of TBI, existence of chronic liver and/or kidney disease, presence of markers of oxidative stress, history and testing of microbial infections (e.g. HSV1), sleep disturbance, testing of BBB leakage in CSF, and exposure to environmental toxins and metal ions. Such a highly integrated composite score would likely provide a more accurate and precise assessment of the risk of developing AD or other dementia in the general population. In addition, it would be beneficial to assess whether this augmented composite score would correlate with observed disease trajectory in individual patients, which is a very valuable piece of information for clinical care.

Another advantage of integrating all conditions listed in section D in the medical history of patients is the possibility to develop personalized medicine approaches in order to prevent and/or reverse AD at early stages of the disease. Clinical interventions would consist of modifying patient’s exposure to environmental toxins and metal ions, and/or adjusting patient lifestyles according to the condition(s) they suffer. As more risk factors for AD are regularly added to the list by panels of expert clinicians [1], the ultimate objective would be to increase the blood flow in the brain in order to improve the physiological perfusion of oxygen and nutrients and remove metabolic waste, thus improving the function of stressed neurons in order to return to a CNS microenvironment as close as possible to homeostasis. Based on the conditions described in section D, several interventions could be proposed: (i) since brain Aβ seems to equilibrate with plasma Aβ, the monitoring of Aβ blood levels with methods like mass spectrometry [194] or immunomagnetic reduction [195] might help determining whether an intervention is necessary to lower systemic Aβ levels; (ii) encouraging moderate-intensity aerobic and resistance exercise in order to stimulate cardiovascular performance, liver metabolism and blood flow in the entire body, though rigorous clinical trials need to be develop to test thoroughly this hypothesis [196]; (iii) when moderate physical exercise is difficult to administer (e.g. disabled patients), considering the possibility to use anti-Aβ therapies such as passive immunotherapies or dialysis [197]; (iv) supplementing patients’ diet by adding a blend of vitamins and minerals (e.g. B12, vitamins A, E, C, Mg^2+^, and Ca^2+^), antioxidants (e.g. N-acteyl-cystein), and omega 3 (e.g. high quality fish oil products) to reduce overall oxidative stress, peripheral chronic inflammation, and improve cellular functions systematically; (v) helping patients to control cholesterol levels by diet adjustments and/or medications to manage atherosclerosis; (vi) controlling diabetes by self-administration of insulin and encouraging better diet; (vii) correcting insulin resistance and glucose hypometabolism identified by FDG-PET [198] by applying interventions like low carbohydrate diets or type-2 diabetes mellitus drugs [163, 172]; (viii) avoiding food and drinks rich in artificial sweeteners like high fructose corn syrup to improve liver functions; (ix) increasing the consumption of vegetables and fruits to increase the overall levels of antioxidants; (x) encouraging patients to quit smoking to improve blood oxygen concentrations, decrease carbon monoxide and dioxide blood levels, and to reduce the detoxification of tobacco-associated toxins by the liver; (xi) encouraging patients to quit alcohol to improve liver function and decrease BBB leakiness; (xii) discussing stress reduction methods with patients to better manage stressful events in their daily life; (xiii) administering antibiotics and encouraging vaccination when appropriate to reduce brain pathogen exposure; (xiv) identifying the cause(s) of sleep disturbances and proposing options to improve sleep at night; (xv) reducing the number of medications to the minimum possible to improve liver functions; (xvi) decreasing the exposure to environmental toxins and metal ions, for example by wearing high quality protective equipment when working in chemical plants and by consuming water filtered for metal ions; and (xvii) administering anti-inflammatory or immunomodulatory medications to manage both systemic and central inflammation (e.g. our group is currently testing immunomodulators in amnestic MCI subjects [199]).

## 5. Conclusion

The careful examination of laboratory AD models and human neuropathology suggests that Aβ plays a very early and important role in responding to disruptions to homeostasis in the CNS and blood vessel endothelium. Contrary to the idea advanced by the amyloid cascade hypothesis, Aβ may be protective for CNS neurons at early stages of stressing events. Only when chronic stressors persist does Aβ become overexpressed and form toxic oligomers and protofibrils. Thus, we provocatively advance that the root cause of AD are pathophysiological changes taking place in the CNS environment that induce chronic neuronal stress. We substantiate our novel theory of multipathology convergence to chronic neuronal stress by presenting a large number of possible clinical conditions that can induce stress in the CNS, all of which are supported by scientific observations. In addition, our original comprehensive theory on AD etiology integrates seamlessly theories proposed previously and it possibly explains the heterogeneity in disease trajectory in individual patients suffering from AD, which would depend upon the combination of underlying co-morbidities. Our view is in full agreement with the earlier opinion devised by Dr. Roses that AD should be considered as a spectrum of diseases rather a single disease [200], and provides a scientific rational for heterogeneous neuropathology. For example, some forms of dementia have been classified as “plaque-only”, “tangle-only”, and some, commonly referred to as “dementia of unknown etiology” (DUE), which show neither plaques, nor tangles, nor other neuropathological feature prototypical of AD [201]. Careful analysis of DUE subjects have suggested that this type of dementia is generally mild in nature and is more prominent in older patients compared to typical AD, with post-mortem pathological features including hippocampal sclerosis and leukoencephalopathy [202]. Such observations could be explained by the stressing of neurons strong enough to perturb CNS homeostasis but without inducing a sustained Aβ overexpression and subsequent plaque formation, thus very similar to single TBI where Aβ is transiently expressed without agglomerating into senile plaques. These types of atypical AD neuropathologies also confirm our recommendation to gather the full history for the medical conditions listed in the present paper to identify which co-morbidity could accompany DUE cases. Finally, our theory opens the possibility of developing targeted therapeutic paradigms using a personalized medicine approach based on each patient’s thorough medical history and risk factors, if applied at an early stage of AD, preferably before dementia manifests. If confirmed, our theory would support the conviction that developing public health strategies to improve healthy aging overall could help lowering the incidence of both heart and neurological diseases [203, 204].
